# Personal Interest Attention Graph Neural Networks for Session-Based Recommendation

**DOI:** 10.3390/e23111500

**Published:** 2021-11-12

**Authors:** Xiangde Zhang, Yuan Zhou, Jianping Wang, Xiaojun Lu

**Affiliations:** College of Sciences, Northeastern University, Shenyang 110819, China; zhangxiangde@mail.neu.edu.cn (X.Z.); 1970039@stu.neu.edu.cn (Y.Z.); wangjianping@mail.neu.edu.cn (J.W.)

**Keywords:** session-based recommendation, graph neural networks, attention, recommendation system

## Abstract

Session-based recommendations aim to predict a user’s next click based on the user’s current and historical sessions, which can be applied to shopping websites and APPs. Existing session-based recommendation methods cannot accurately capture the complex transitions between items. In addition, some approaches compress sessions into a fixed representation vector without taking into account the user’s interest preferences at the current moment, thus limiting the accuracy of recommendations. Considering the diversity of items and users’ interests, a personalized interest attention graph neural network (PIA-GNN) is proposed for session-based recommendation. This approach utilizes personalized graph convolutional networks (PGNN) to capture complex transitions between items, invoking an interest-aware mechanism to activate users’ interest in different items adaptively. In addition, a self-attention layer is used to capture long-term dependencies between items when capturing users’ long-term preferences. In this paper, the cross-entropy loss is used as the objective function to train our model. We conduct rich experiments on two real datasets, and the results show that PIA-GNN outperforms existing personalized session-aware recommendation methods.

## 1. Introduction

In recent years, the rapid development of Internet technology in many applications (such as e-commerce, social media, etc.) has caused information overload. Recommendation system play a crucial role in helping users alleviate information overload and select interested information. Among them, content-based RS and CF-based RS are the two most widely used methods which make simple and efficient recommendations by calculating the similarity between items. These methods assume that all historical interactions of users are equally important to their current preferences, but this is impossible in reality. The user’s click depends not only on their long-term preference, but also on their short-term preference and time-sensitive context. In many cases, the items currently interacting with the user can better reflect their recent preferences. For example, in e-commerce, the user is more likely to purchase recently viewed products because these products are more representative of the user’s recent needs. In this case, content-based RS and collaborative filtering-based RS fail to capture the change of user’s preferences and recommend inaccurately. In addition, most existing recommendation systems use users’ information and historical behavior records for personalized recommendation. However, in actual applications, user information is unknown, and user history behaviors are not available besides the current session, so it is difficult to produce accurate results. To solve these problems, a session-based recommendation system extracts interactive information to represent the user’s preference transfer and use the limited historical behavior to predict the user’s next action (such as which item to click).

The internet contains a large amount of information, and users’ interests are constantly changing. A session-based recommendation system can capture a user’s short-term preferences from a user’s recently generated session and get the changes of users’ preferences between different sessions. Based on the above two points, session-based recommendation system is widely used in shopping and short video APPs.

Since the session sequence is divided by time, it can be represented as time series, so Markov chains can be used. Markov assume that the user’s next action is only influenced by the current action, regardless of other factors. This strong independence assumption is vulnerable to noisy data and may limit the accuracy of recommendations. The research of [1] showed that user preferences are not entirely dependent on the time-order of sequences and emphasized the importance of recurrent neural networks (RNNs) in a session-based recommendation system. Recent methods divide user preferences into long-term preferences and short-term preferences. Hidasi et al. proposed an RNN-based method GRU4Rec [2] which models user short-term preferences by encoding items. NARM [3] uses two RNN-based subsystems to capture the long-term and short-term preferences of users respectively. This method selects the last item in the session to represent short-term preferences, and others represent long-term preferences. Similar to NARM, STAMP [4] uses a simple MLP model and an attentive net to extract users’ potential interests. Although these methods have achieved good results, we believe that these methods are still in their infancy and have certain limitations. Complex user behavior patterns are important for session-based recommendation, yet the above sequence-based approach only modeled sequential transitions between consecutive items, ignoring the relationship between other items. To overcome this limitation, this paper models the items in a session as a session graph, captures the transitions between items by GNN, and generates an embedding vector representation.

In addition, when making recommendations, the candidates are rich and the users’ interests are usually diverse. For example, as a boy, Alex is interested in computers, sneakers, and game consoles at the same time. Therefore, over a period of time, he may click on items from all three categories. Many recent models [3,4,5] represent user interests as a fixed-size embedding vector, which cannot express multiple interests of users, and limit the expressiveness of recommendation models. It is not necessary to embed all user interests into a single vector when making predictions for a specific candidate. For example, suppose Alex has a historical session (computer, sneakers, milk, game console). If we want to recommend gamepads to him, we will focus on his interest in game consoles rather than milk. In other words, we can recommend items based on user behavior for a user’s specific interests.

**Figure 1:**
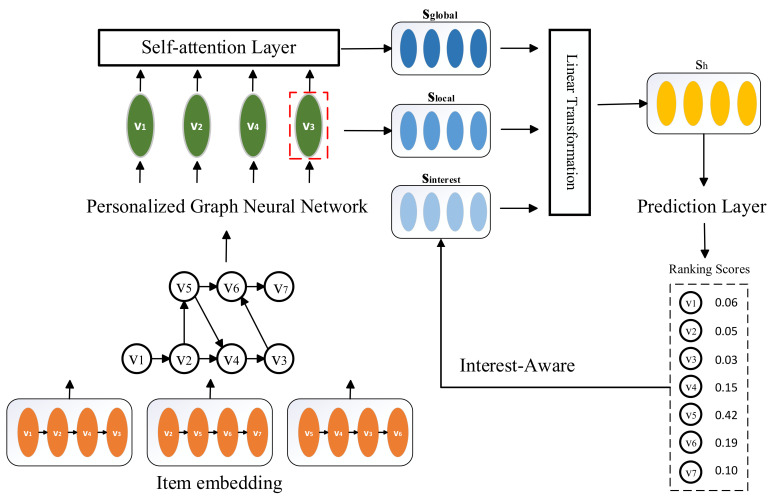
Workflow of the PIA-GNN method. We model all sessions as session graphs and process each session graph one by one. Then a node vector representation is obtained using PGNN network. The global embedding $s_{global}$ is obtained using the self-attention mechanism, the last item of the session is used as the local embedding $s_{local}$, and an interest embedding $s_{interest}$ is obtained by interest perception. In the prediction layer, we represent each session as a linear representation of the interest embedding, local embedding and global embedding. Finally, we calculate the recommendation ranking score for each candidate item.

Figure 1 illustrates the workflow of the proposed PIA-GNN method. In this paper, we generate a representation for each item by modeling session sequences as graph structures data that capture the complex transitions between items [6,7]. We use a personalized graph neural network (PGNN) to further strengthen the association between each user and its different sessions by adding users’ information when updating the node embedding updates. In addition, in order to analyze the specific interests of the user, we add a new interest attention module, which adaptively activates user interest by considering the historical behavior correlation of a given target item. Then, we use the self-attention network to obtain the global embedding. Finally, we use the user’s target, global and local embedding in the session to construct the session embedding, and infer the user’s next action based on the item embedding and session embedding.

Main contributions:

- For personalized recommendation scenarios, we use a new user-based personalized graph neural network (PGNN), which can capture complex item transformations for different user interests.
- For the different interests of users, we add the interest attention module, which can activate different user interests adaptively for different targets and improve the expressiveness of the model.
- We have studied the model on two real-world datasets. Experiments demonstrate the effectiveness of the proposed model.

## 2. Relate Work

In this section, we review some related work on session-based recommender systems, including traditional approaches, sequential approaches based on Markov chains, and RNN-based approaches. Then, we introduce neural networks on graphs.

### 2.1. Traditional Recommendation Method

Matrix factorization [8,9,10] is a frequently method used in recommender systems. MF factorize a user-item evaluation matrix into two low-rank matrices, each of which represents the potential factors of a user or an item. In matrix factorization, user preferences can only be provided by some positive click behaviors and are not applicable to session-based recommendations. In item-based neighborhood methods [11], item similarity can be calculated by all items in a session. However, these methods have difficulty in considering the order of items and generate predictions based on the last click only. To solve the above problem, Markov chain-based sequential prediction methods are proposed, which predict the user’s next behavior based on the last one. Zimdars et al. [12] used the probabilistic decision tree model to encode the conversion mode of goods. Shani et al. [13] regard recommendation generation as a sequential optimization problem, which is solved by Markov decision processes (MDPs). FPMC [14] models the sequence behavior between two clicks by decomposing the user’s personalized probability transfer matrix and provide prediction for each sequence more accurately. The main drawback of Markov chain-based models is that they independently combine past components. This independence assumption is too strong and limits the accuracy of the predictions.

### 2.2. Deep-Learning-Based Methods

In recent years, deep neural networks have been successfully applied to machine translation [13,15,16], conversational machines [17], and other sequential modeling fields and achieved good results. For session-based sequential recommendation, a recurrent neural network approach was proposed in [2]. On this basis, Hidasi et al. [18] proposed a model, GRU4REC, that applies RNN networks to SBR, which uses multilayer gated recurrent units (GRUs) to model item interaction sequences and can model conversations based on the characteristics of clicked operations and clicked items. Tan et al. [9] improved the performance of recurrent model by considering temporal changes in user behavior and appropriate data augmentation techniques. The NARM model [3] integrates the attention mechanism into the GRU encoder and designs a global and local RNN recommender to capture users’ sequential behavior and primary purpose. The SHAN model [19] uses a two-layer hierarchical attention network that considers both long-term and short-term preferences. Liu et al. [4] proposed an attention-based short-term memory network (STAMP), using a simple MLP network and an attention network to capture the general and current interests of users. Both NARM and STAMP use an attention mechanism to emphasize the importance of the last click. However, the above session or sequence-based models can only make suggestions using current anonymous sessions or individual sequences.

### 2.3. Graph Neural Networks

Nowadays, neural networks have been used to generate representations of graph-structured data, such as social networks and knowledge bases. On the one hand, the unsupervised algorithm Deep Walk [20], extending word2vec [21], learns graph node representations by random walk. Following Deep Walk, the unsupervised network embedding algorithms LINE [22] and node2vec [23] are the most representative approaches. On the other hand, the classical neural networks CNN and RNN are also applied on graph structured data. Duvenaud et al. [24] introduced a convolutional neural network that can directly operate on graphs of any sizes and shapes. Thomas N [25] selected the convolution architecture by a localized approximation of spectral graph convolution, which is an effective variant that can directly operate on the graph. However, these methods can only be used for undirected graphs. Previously, graph neural networks (GNNs) [26,27] were proposed in the form of recurrent neural networks to operate on directed graphs. GNNs are good at processing graph-structured data and can capture richer information in sequential data. Gated graph neural networks [28] use gated recurrent units and back propagation in time (BPTT) to compute gradients based on GNNs. Graph attention networks (GAT) [29] uses an attention mechanism to learn the weights of nodes and neighbor nodes. Wu et al. [12] proposed a gated GNN model (called SR-GNN) to learn item embeddings on the session graph, and then integrated each learned item embedding based on the attention value to obtain a representative session embedding, and the attention value is calculated based on the relevance of each item to the last item. SR-GNN [12] is the first model that captures complex item transition relationships using gated graph neural networks in a session-based recommendation scenario, but it ignores the user’s role in item transition relationships and does not exploit historical user session information to improve recommendation performance. In this paper, we propose the PTA-GNN model that can make personalized session recommendations for user-specific interests.

## 3. The Proposed Method

In this section, we introduce the proposed PTA-GNN model, that uses personalized graph neural network, user interest perception embedding and attention mechanism to achieve session-based recommendation. We describe the problem first. Then, the proposed model is presented in this part.

### 3.1. Problem Statement

Session-based recommendation aims to predict which item the user wants to click next based on anonymous sessions. Here, we give the formulation of the session-based problem as follows. In session-based recommendation, let $V=\left\{v_{1},v_{2},\cdots ,v_{\left|V\right|}\right\}$ represent the set of all unique items involved in all sessions. For each anonymous session s, the sequence of actions clicked by the user is denoted as $s=\left\{v_{s,1},v_{s,2},\cdots ,v_{s,n}\right\}$, where $v_{s,i}\in V$ is the item clicked by the user within the session and n stands for the total number of sessions for a user u. Given the user’s action sequence $s=\left\{v_{s,1},v_{s,2},\cdots ,v_{s,n}\right\}$, our model aims to predict the next click $\left(v_{s,n+1}\right)$. To be precise, our model generates a ranking list of all the candidates that may appear in the session as $\hat{y}=\left\{{\hat{y}}_{1},{\hat{y}}_{2},\cdots ,{\hat{y}}_{\left|V\right|}\right\}$ represents the output probability of all items, where $\;{\hat{y}}_{i}$ represents the recommendation score of the item $v_{i}$. Since recommenders usually recommend users more than once, we select the Top−k items from $\hat{y}$ for recommendation.

### 3.2. Constructing Session Graphs

**Figure 2:**
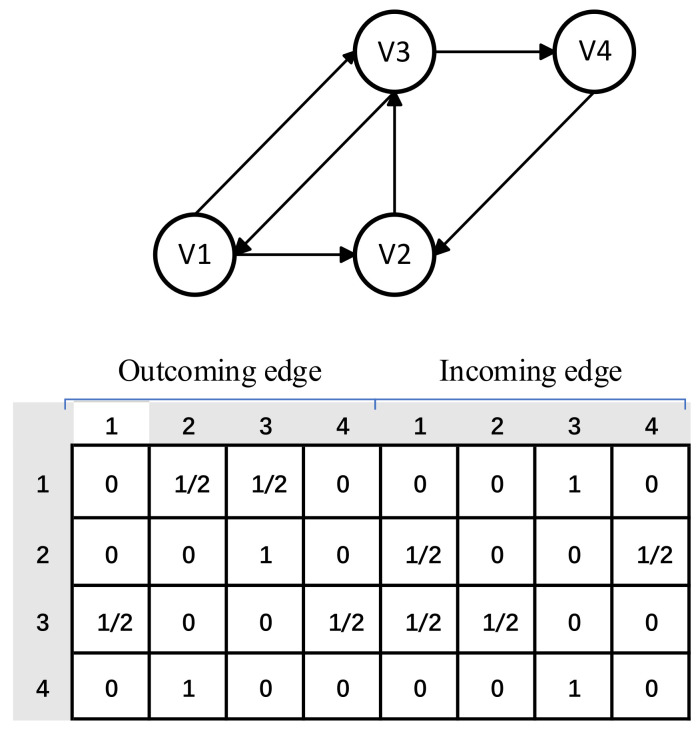
An example of a session graph and the connection matrix $A_{s}$.

Each session sequence s can be modeled as a directed graph $G_{s}=\left(V_{s},E_{s}\right)$, where $V_{s}$ is the set of nodes and $E_{s}$ is the set of edges, in this session graph $G_{s}$, each node represents an item $v_{s,i}\in V$. Edge $\left(v_{s,i-1},v_{s,i}\right)\in E_{s}$ represents that the user successively clicks the item $v_{s,i-1}$ and the item $v_{s,i}$. Therefore, each session sequence can be modeled as a directed graph. Since the items in the sequence may be repeated, we assign a normalized weight to each edge, which is the number of occurrences of the edge divided by the outgoing degree of the node where the edge starts. In the session graph, the edge $v_{i}\to v_{j}$ represents that the user first clicked on item $v_{i}$ and then clicked on item $v_{j}$ in a particular session. In this case, we assume that on the edge $v_{i}\to v_{j}$, the effect of $v_{i}$ on $v_{j}$ and the effect of $v_{j}$ on $v_{i}$ are different, and it produces two types of edges to represent two different transformation relationships. These two directed edges are called the incoming and outgoing edges, and the weights are $\omega_{ij}^{in}$ and $\omega_{ij}^{out}$, respectively. The weights can be calculated by Equations (1) and (2) as follows:

(1) $$\omega_{ij}^{in}=\frac{Count\left(v_{j},v_{i}\right)}{\sum_{v_{k\to }v_{i}}Count(v_{k},v_{i})}$$

(2) $$\omega_{ij}^{out}=\frac{Count\left(v_{i},v_{j}\right)}{\sum_{v_{i\to }v_{k}}Count(v_{i},v_{k})}$$

(3) $$A_{s}^{in}\left[i,j\right]=\omega_{ij}^{in}$$

(4) $$A_{s}^{out}\left[i,j\right]=\omega_{ij}^{out}$$

where the function $Count\left(x,y\right)$ is used to count the number of times the user interacts with the item y after interacts with the item x. $A_{s}$ is defined as the connection matrix of two adjacency matrices $A_{s}^{out}$ and $A_{s}^{in}$, where $A_{s}^{out}$ and $A_{s}^{in}$ denote the weighted connections of outgoing and incoming edges in the session graph. As in Figure 2, for a session $s=\left\{v_{1},v_{2},v_{3},v_{1},v_{3},v_{4},v_{2}\right\}$, we can construct a session graph $G_{s}$ and a matrix $A_{s}$. Here, we use the same strategy for constructing session graphs as SR-GNN. Note that for session graphs with different structures, PTA-GNN can support various strategies for constructing session graphs and generating the corresponding connection matrices to update the graph structure by facilitating the information transfer between different nodes. We embed each item v∈V into a uniform embedding space, and the node vector $\nu \in {\mathbb{R}}^{d}$ denotes the latent vector of item v learned through the graph neural network, where d is the dimension. Based on the node vectors, each session s can be represented by an embedding vector, which consists of all node vectors in the graph.

### 3.3. Personalized Graph Neural Network

The application of GNN to session-based recommendation was first proposed in the SR-GNN model, which provides a session graph containing rich node connections to GGNN to automatically extract useful features of items. However, SR-GNN does not inject user information into the graph model, so it is not suitable for personalized recommendation to users. To solve this problem, we use the personalized graph neural networks (PGNN) to learn complex transformational relationships between items that have interacted with the user, and then obtain the representations of items and users.

Different users have different behavior patterns, which leads to different project conversion relationships for each user. Therefore, we consider user factors when designing the PGNN architecture. At each node update, we contact the user embedding $e_{u}$ and the current representation of the node $v_{i}^{t-1}$. At moment t, the aggregated information of input and output of node i is represented as follows:

(5) $$a_{in_{s,i}}^{\left(t\right)}=\underset{v_{j}\to v_{i}}{\sum }A_{s}^{in}\left[i,j\right][v_{j}^{\left(t-1\right)}∥e_{u}]W_{in}$$

(6) $$a_{out_{s,i}}^{\left(t\right)}=\underset{v_{i}\to v_{j}}{\sum }A_{s}^{out}\left[i,j\right][v_{j}^{\left(t-1\right)}∥e_{u}]W_{out}$$

(7) $$a_{s,i}^{\left(t\right)}=a_{out_{s,i}}^{\left(t\right)}∥a_{in_{s,i}}^{\left(t\right)}$$

where ∥ is the connection operation, and since the session graph $G_{s}$ is a directed graph, we use two parameters $W_{in}$ and $W_{out}\in R^{\left(d+d^{\prime }\right)\times \hat{d}}$ to convert the user and item connection vectors into two different $\hat{d}$ dimensional vectors.

Then, we use gated recurrent units (GRUs) to merge the hidden state information from the previous time step of other nodes and update the hidden state of each node:

(8) $$z_{s,i}^{t}=\sigma \left(W_{z}a_{s,i}^{t}+U_{z}v_{i}^{t-1}\right)$$

(9) $$r_{s,i}^{t}=\sigma \left(W_{r}a_{s,i}^{t}+U_{r}v_{i}^{t-1}\right)$$

(10) $$\tilde{v_{i}^{t}}=\tanh\left(W_{o}a_{s,i}^{t}+U_{o}\left(r_{s,i}^{t}⊙v_{i}^{t-1}\right)\right)$$

(11) $$v_{i}^{t}=\left(1-z_{s,i}^{t}\right)⊙v_{i}^{t-1}+z_{s,i}^{t}⊙\tilde{v_{i}^{t}}$$

where $\sigma \left(\cdot \right)$ is the sigmoid function and ⊙ is the element multiplication operator, $W_{z},U_{z},W_{r},U_{r},W_{o},U_{o}$ are the parameters of GRU, which are shared by all users. Parameter $r_{s,i}^{t}$ is the forgetting gate, which controls the forgotten information, and $z_{s,i}^{t}$ is the updating gate, which controls the newly generated information. The $\left(1-z_{s,i}^{t}\right)$ in Equation (11) selects which past messages are forgotten and $z_{s,i}^{t}$ selects which newly generated messages are remembered. The $r_{s.i}^{t}$ in Equation (9) determines from which past information new information is generated. Parameter $\tilde{\;v_{i}^{t}}$ is the new information generated, and $v_{i}^{t}$ is the final updated node state.

Similar to most graph-based models [5,30], PGNN is suitable for scenarios where users repeatedly click on the same items within or across sessions. In this scenario, all sessions of that user can be converted into a fully connected graph structure, and thus, PGNN can capture item conversion patterns across sessions. When there are no duplicate items in multiple sessions, the user’s behavior graph contains many disconnected subgraphs, each of which corresponds to a single session. Since all sessions of each user share the same user embedding, each subgraph in the user’s behavior graph can be associated by user embedding when the node embedding is updated, and cross-session associations can be captured.

### 3.4. Constructing Interest-Aware Embeddings

Previous work used in-session items to capture users’ interests. In this section, we construct user interest embeddings to adaptively consider the relevance of the user’s items of interest and the user’s historical behavior. We define interest items as all candidates to be predicted. Normally, the user’s operation only matches a part of his interest, and to simulate this process, we design a new interest-attention mechanism to compute the softmax score for each target item in the session.

(12) $$\beta_{i,t}=softmax\left(e_{i,t}\right)=\frac{\exp\left(v_{t}^{T}Wv_{i}\right)}{\sum_{j=1}^{n}\exp\left(v_{t}^{T}Wv_{i}\right)}$$

Finally, for each session s, the user’s interest in the target item $\;v_{t}$ can be expressed as $s_{interest}^{t}\in {\mathbb{R}}^{d}$, computed as follows:

(13) $$s_{interest}^{t}=\overset{s_{n}}{\underset{i=1}{\sum }}\beta_{i,t}v_{i}$$

For different target projects, we can get different interest embedding from users.

### 3.5. Self-Attention Layers

Recently, a new sequential model, Transformer [31], has achieved state-of-the-art performance and efficiency in various translation tasks. Transformer employs a multilayer self-attentive network that fully considers all signals using a weighted average operation. The self-attentive model, as a special attention mechanism, has been widely used to model sequential data and has achieved significant results in machine translation [31], sentiment analysis [3], and sequential recommendation [32,33]. The self-attention mechanism can map the global dependencies between inputs and outputs and capture the item-item transitions across the input and output sequences without considering the distance between them.

The input of Transformer attention consists of queries and keys of dimension $\sqrt{d_{k}}$ and values of dimension $\sqrt{d_{v}}$. To stabilize the gradient, transformer uses score normalization, which is divided by $\sqrt{d_{k}}\;$, then use the softmax function to obtain the weight values. The scaled dot-product attention is formally defined as:

(14) $$E=softmax\left(\frac{\left(VW^{Q}\right){\left(VW^{K}\right)}^{T}}{\sqrt{d}}\right)\left(VW^{V}\right)$$

where the projection matrices $W^{Q},W^{K},W^{V}\in {\mathbb{R}}^{2d\times d}$, Q,K,V represent Query, Key, and Value respectively.

#### 3.5.1. Point-Wise Feed-Forward Network

We apply two linear transformations and a ReLU activation function to endow the function with nonlinearity and consider the interactions between different dimensions. Nevertheless, information transfer loss occurs during the self-attentive operation, so we add residual connections after the feedforward network to make the model more easily exploit the underlying information:

(15) $$G=ReLU\left(EW_{1}+b_{1}\right)W_{2}+b_{2}+E$$

where $W_{1},W_{2}$ are d×d-dimensional matrices and $b_{1},b_{2}$ are d-dimensional bias vectors. To alleviate the overfitting problem of deep neural networks, we use the “Dropout” regularization technique to randomly discard data during training. We define the above self-attentive mechanism as follows:

(16) $$G=SAN\left(V\right)$$

#### 3.5.2. Multi-Layer Self-Attention

Recent studies have shown that different layers can capture different types of features. In this work, we studied which layers that benefit most from feature modeling to learn more complex project conversion. The first layer is defined as $G^{\left(1\right)}=G$ and the $k\;\left(k>1\right)$ layer is defined as:

(17) $$G^{\left(k\right)}=SAN\left(G^{\left(k-1\right)}\right)$$

where $G^{k}\in {\mathbb{R}}^{n\times d}$ is the final output of the multi-layer self-attention networks.

### 3.6. Generating Session Embeddings

To predict the user’s next click better, we plan to develop a strategy that combines the long-term and short-term preferences of the session with the user’s interest-aware embedding, and embed this combination as session embedding.

Local embedding: Since the user’s behavior in the next moment is usually determined by the behavior in the current moment. For session $s=\left[v_{s,1},v_{s,2},\cdots ,v_{s,n}\right]$, we simply define local embedding as the last-clicked item $v_{s,s_{n}}$ accessed by the user in the current session s, i.e., $s_{local}=v_{s,n}$.

Global embedding: Then we represent the user’s long-term preferences as a global embedding $s_{global}\in {\mathbb{R}}^{d}$, and we take the last dimension of $G^{k}$ as the global embedding vector, i.e., $s_{global}=G^{k}$.

Session embedding: Finally, we generate session embeddings for session s by linearly transforming the local and global embeddings as well as the tandem of interest embeddings.

(18) $$s_{h}=W_{3}\left[s_{interest}^{t}∥s_{local}∥s_{global}\right]$$

where matrix $W_{3}\in {\mathbb{R}}^{d\times 3d}$ compresses three combined embedding vectors into the latent space ${\mathbb{R}}^{d}$, and it is worth noting that we generate different session embeddings for different items of interest.

### 3.7. Making Recommendation and Model Training

After obtaining the embedding of each session, we calculate the recommendation score $\hat{z_{i}}$ for each candidate item $v_{i}\in V$ by the inner product of each item embedding $v_{i}$ and the session embedding $s_{h}$. After that, we normalize the scores $\hat{z_{i}}$ for all target items using the softmax function and obtain the final output vector.

(19) $$\hat{z_{i}}=s_{h}^{T}v_{i}$$

(20) $$\hat{y}=softmax\left(\hat{z}\right)$$

where $\hat{z}\in {\mathbb{R}}^{m}$ represents the recommendation score for all candidates and $\hat{y}\in {\mathbb{R}}^{m}$ represents the probability of a node appearing in session s and being clicked on next time.

For each session graph, the loss function is defined as the cross-entropy of the predicted value and the ground truth. We train our model by minimizing the following objective function:

(21) $$L\left(\hat{y}\right)=-\underset{i=i}{\sum }y_{i}\log\left(\hat{y_{i}}\right)+\left(1-y_{i}\right)\log\left(1-\hat{y_{i}}\right)$$

where y represents the one-hot encoding of the ground truth item.

Finally, we use the backpropagation through time (BPTT) algorithm to train our model. Since in the session-based recommendation scenario the length of most sessions is short, we try to choose as few training steps as possible to prevent overfitting.

## 4. Experiment and Analysis

In this section, we present the dataset, comparison methods, and evaluation metrics used in the experiments. Then, our proposed PIA-GNN model is compared with other methods.

### 4.1. Datasets

We evaluate the proposed approach on two widely used real datasets, Yoochoose and Diginetica. The Yoochoose dataset comes from the 2015 data mining conference RecSys Challenge, which contains user click information from an e-commerce website over a six-month period. The Diginetica data come from the 2016 CIKM Challenge Cup, which uses only the user’s transaction information.

For a fair comparison, we use the data preprocessing scheme of Li et al. [3], Liu et al. [4], and Wu et al. [5]. We discarded items with less than five occurrences in both datasets and sessions with session lengths no greater than one. The remaining 7,981,580 sessions and 37,483 items constitute the Yoochoose dataset, and 204,771 sessions and 43,097 items constitute the Diginetica dataset. To generate the training and test sets, Yoochoose uses the last few days of sessions as the test set and Diginetica uses the last few weeks of sessions as the test set. For example, for an existing session $s=\left[v_{s,1},v_{s,2},\cdots ,v_{s,n}\right]$, we generate a sequence of input sessions and the corresponding labels:

(22) $$\left(\left[v_{s,1}\right],v_{s,2}\right),\left(\left[v_{s,1},v_{s,2}\right],v_{s,3}\right),\cdots ,\;\left(\left[v_{s,1},v_{s,2},\cdots ,v_{s,n-1}\right],v_{s,n}\right)$$

where $\left[v_{s,1},v_{s,2},\cdots ,v_{s,n-1}\right]$ represents the generated sequence and $v_{s,n}$ represents the next clicked item, i.e., the label of the sequence. Since the Yoochoose dataset is too large, we take its nearest 1/64 training part, denoted as Yoochoose 1/64. The statistical results of all the datasets applied in the experiment are shown in Table 1.

### 4.2. Baseline

To evaluate the performance of the proposed method, we compare it with the following representative baseline.

- POP always recommends the most popular program in the entire training set, and despite its simplicity, it can serve as a powerful baseline in certain situations.
- S-POP recommends the top TOP-N most popular items for the current session.
- Item-KNN [27] recommends items that are similar to the items clicked in previous sessions, where similarity is defined as the cosine similarity between session vectors. Regularization is introduced to avoid the high similarity problem between unvisited items.
- BPR-MF [34] proposed a BPR objective function to compute pairwise ranking losses, using stochastic gradient descent to optimize the pairwise ranking objective function. The matrix decomposition is applied to session-based recommendations using the average potential vector of items in a session.
- FPMC [14] is a Markov chain-based sequence prediction method.
- GRU4REC [2] uses RNNs to model user sequences for session-based recommendations, stacking multiple GRU layers to encode session sequences into a final state. A ranking loss is used to train the model.
- NARM [28] uses RNN with attention mechanism to capture the main purpose and sequential behavior of the user.
- STAMP [4] uses the attention layer to replace the RNN encoder, and even completely relies on the self-attention of the last item in the sequence to make the model more powerful.
- SR-GNN [5] uses the gated graph convolutional layer to obtain the embedding of all items, and then it pays attention to each last item like STAMP to calculate the sequence-level embedding.

### 4.3. Evaluation Metrics

At each recommendation, a recommender system can give several recommended items, and the user will select the top ones of them. In order to keep the same setup as the previous baseline, we mainly chose to use top20 items to evaluate the recommender system, specifically, using two metrics, Recall@20 and MMR@20.

Recall@ (The number of recalls is calculated by the first Top-K). Recall is the ratio of the number of correct items to the number of items in the test set for the top K items in the recommendation ranking, i.e.,

(23) $$Recall@K=\frac{n_{hit}}{N}$$

where N represents the number of test sequences $S_{test}$ in the data and $n_{hit}$ represents the number of items needed among the top K items in the ranking.

MMR@K (Mean inverse ranking) is the average of the inverse of the ranking of the correct recommendation. When the ranking exceeds 20, the reciprocal value takes 0. MRR measures the order of the recommendation ranking, and a higher MMR value means that the correct recommendation is at the front of the ranking list.

(24) $$MRR@K=\frac{1}{N}\underset{v_{label}\in S_{test}}{\sum }\frac{1}{Rank\left(v_{label}\right)}$$

### 4.4. Parameter Setting

Following the previous approach [3,4], we made the potential vector dimension d=100 for all data sets. In addition, we selected other hyperparameters on a 10% random validation set. All parameters were initialized using a Gaussian distribution with a mean of 0 and a standard deviation of 0.1. A small-batch Adam optimizer was used to optimize these parameters, and the initial learning rate was set to 0.001, decaying by 0.1 after every 3 epochs. In addition, the batch size and L2 penalty were set to 100 and $10^{-5}$, respectively.

### 4.5. Comparison with Baseline Methods

To evaluate the performance of the proposed method, we first compare it with existing representative baselines. The performance of Recall@20 and MMR@20 is summarized in Table 2, where the best performance is highlighted in bold.

PIAGNN aggregates session items into the session graph, merges user embeddings to increase the personalized representation of the model, and further considers modeling user preferences through their interest-aware attention. As can be seen from the table, the proposed PIAGNN model achieves state-of-the-art performance on all datasets of Recall@20 and MRR@20, which confirms the effectiveness of the proposed approach.

For traditional popularity-based algorithms such as POP and S-POP, the performance is relatively poor on both datasets. These simple models make recommendations based only on repeated identical items or consecutive items, which is problematic in a session-based recommendation scenario. Nevertheless, S-POP still outperforms methods, such as POP, BPR-MF, and FPMC, which illustrates the important impact of contextual information on recommendations in a session. BPR-MF improves performance compared to POP by analyzing users individually and optimizing the pairwise ranking loss function, which reflects the importance of user personalization in recommendations. Item-KNN achieves the best results, but Item-KNN only uses the similarity between items to recommend items with high similarity without considering the order information, which may be because the underlying factors representing user preferences play a key role in the recommendation. It can be seen that Item-KNN outperforms most Markov chain-based methods, which indicates that the assumptions about the independence of consecutive items, on which the traditional MC-based approach relies, are unrealistic in the context of a session-based recommendation scenario.

Compared to the traditional approaches described above, neural network-based approaches have a greater ability to capture complex user behaviors and therefore offer a significant performance improvement. Long-term and short-term memory models, such as GRU4REC and NARM, use recurrent units to capture users’ general interests. These methods model users’ global preferences and consider transitions between users’ previous actions and the next click, thus improving the performance of the models. STAMP performs better than GRU4REC by using the last-clicked item as a short-term memory, which shows the effectiveness of short-term behavior in predicting the next clicked item. SR-GNN further modeled sessions as session graphs and applies graph neural networks and attention mechanisms to capture complex item transitions, outperforming other baselines on both datasets. However, the performance of these models is still inferior to that of the proposed approach. PIAGNN incorporates user embeddings to improve the personalized representation of the model, uses goal-aware attention to explore user interest preferences to enrich the graph-based model, and activates different user interests for different target items to improve the expressiveness of the recommendation model. In addition, the attention mechanism is used to adaptively capture the long-term dependencies between session items. Taken together, these results demonstrate the effectiveness of the proposed PIAGNN approach.

### 4.6. Ablation Studies

Our approach is next compared with different variables to verify the effectiveness of the PGNN and interest-aware key modules. PIA-GNN(-P): PIA-GNN without user embedding; PIA-GNNN(-I): PIA-GNN without the Interest-aware mechanism, i.e., without considering the user’s interest preferences; PIA-GNN(-U-I): PIA-GNN does not contain either PGNN component or Interest-aware mechanism. The same as the session-based approach. We show the results for Recall@20, MMR@20 in Table 3 and have the following findings.

PIA-GNN (-I) results outperform PIA-GNN (-I-U-A), which illustrates that our personalized graph neural network PGNN is better than GNN in capturing complex transformational relationships between items. PIA-GNN (-P) results outperform PIA-GNN (-I-U-A), which indicates that our interest exploration module can capture users’ interest in specific types of items. PIA-GNN consistently outperforms PIA-GNN (-P) and PIA-GNNN (-I). The importance of personalized graph neural networks and modeling for user-specific interests is illustrated.

## 5. Conclusions

In this paper, we proposed a personalized interest-attention graph neural network, PIA-GNN, based on session recommendation. Using a personalized graph neural network (PGNN), user information is added when node embedding is updated to capture the complex transformation relationships between items. Moreover, an interest-attention module is added to adaptively activate the user’s interest in specific types of items for different targets. In addition, the long-term dependencies between sessions are captured using a self-attention module. The model is trained by minimizing the cross-entropy loss function of the predicted value and ground truth. Subsequently we tested on two real-world datasets corroborates that the PIA-GNN model outperforms other models in most cases. The effectiveness of each component of the model is confirmed by comparison tests.

## Figures and Tables

**Table 1:** Statistics of datasets used in the experiments.

Statistics	Diginetica	Yoochoose 1/64
#Clicks	982,961	557,248
#Training sessions	719,470	369,859
#Test sessions	60,858	55,898
#Unique items	43,097	16,766
Average length	5.12	6.16

**Table 2:** The performance of PIAGNN compared with other baseline methods using two datasets. The best results highlighted in boldface.

Method	Diginetica		Yoochoose 1/64	
	Recall@20	MRR@20	Recall@20	MRR@20
POP	0.89	0.20	6.71	1.65
BPR-MF	5.24	1.98	31.31	12.08
S-POP	21.06	13.68	30.44	18.35
FPMC	26.53	6.95	45.62	15.01
GRU4REC	29.45	8.33	60.64	22.89
Item-KNN	35.75	11.57	51.60	21.81
NARM	49.70	16.17	68.32	28.63
STAMP	45.64	14.32	68.74	29.67
SR-GNN	50.73	17.59	70.57	30.94
PIA-GNN	**52.62**	**18.39**	**71.46**	**31.27**

**Table 3:** The performance of the PIA-GNN and four ablation models on two datasets with the rating.

Method	Diginetica		Yoochoose 1/64	
	Recall@20	MRR@20	Recall@20	MRR@20
PIA-GNN	52.62	18.39	71.46	31.27
PIA-GNN(-U)	51.83	18.26	71.02	31.22
PIA-GNN(-I)	51.28	18.19	70.98	31.08
PIA-GNN(-I-U-A)	50.94	17.85	70.69	30.99

## Data Availability

Data Availability Statement: Publicly available datasets were analyzed in this study. YOOCHOOSE: Available online: http://2015.recsyschallenge.com/challege.html (11 October 2021), DIGINETICA: Available online: https://competitions.codalab.org/competitions/11161(11 October 2021).
